# A Conversation on Data Mining Strategies in LC-MS Untargeted Metabolomics: Pre-Processing and Pre-Treatment Steps

**DOI:** 10.3390/metabo6040040

**Published:** 2016-11-03

**Authors:** Fidele Tugizimana, Paul A. Steenkamp, Lizelle A. Piater, Ian A. Dubery

**Affiliations:** 1 Department of Biochemistry, University of Johannesburg, Auckland Park, Johannesburg 2006, South Africa; fideletu@gmail.com (F.T.); psteenkamp@csir.co.za (P.A.S.); lpiater@uj.ac.za (L.A.P.); 2 Drug Discovery and Development, Biosciences, CSIR, Pretoria 0001, South Africa

**Keywords:** chemometrics, data mining, metabolomics, pre-processing, pre-treatment, scaling, transformation

## Abstract

Untargeted metabolomic studies generate information-rich, high-dimensional, and complex datasets that remain challenging to handle and fully exploit. Despite the remarkable progress in the development of tools and algorithms, the “exhaustive” extraction of information from these metabolomic datasets is still a non-trivial undertaking. A conversation on data mining strategies for a maximal information extraction from metabolomic data is needed. Using a liquid chromatography-mass spectrometry (LC-MS)-based untargeted metabolomic dataset, this study explored the influence of collection parameters in the data pre-processing step, scaling and data transformation on the statistical models generated, and feature selection, thereafter. Data obtained in positive mode generated from a LC-MS-based untargeted metabolomic study (sorghum plants responding dynamically to infection by a fungal pathogen) were used. Raw data were pre-processed with MarkerLynx^TM^ software (Waters Corporation, Manchester, UK). Here, two parameters were varied: the intensity threshold (50–100 counts) and the mass tolerance (0.005–0.01 Da). After the pre-processing, the datasets were imported into SIMCA (Umetrics, Umea, Sweden) for more data cleaning and statistical modeling. In addition, different scaling (unit variance, Pareto, etc.) and data transformation (log and power) methods were explored. The results showed that the pre-processing parameters (or algorithms) influence the output dataset with regard to the number of defined features. Furthermore, the study demonstrates that the pre-treatment of data prior to statistical modeling affects the subspace approximation outcome: e.g., the amount of variation in X-data that the model can explain and predict. The pre-processing and pre-treatment steps subsequently influence the number of statistically significant extracted/selected features (variables). Thus, as informed by the results, to maximize the value of untargeted metabolomic data, understanding of the data structures and exploration of different algorithms and methods (at different steps of the data analysis pipeline) might be the best trade-off, currently, and possibly an epistemological imperative.

## 1. Introduction

Metabolomics, a rapidly developing post-genomic approach, has proven to be a powerful and indispensable tool to interrogate cellular biochemistry, investigating metabolism and its reciprocal crosstalk with cellular signaling and regulation [1,2]. The metabolic profiles may be seen as functional signatures of the physiological state of the biosystem under investigation, i.e., snapshots of partially-mapped molecular landscapes, comprising effects of genetic regulation, as well as environmental factors [3,4,5]. However, the realization of a holistic coverage of the whole metabolome, in a given biological system, is still currently not feasible (at least with a single method) at the metabolite extraction [6,7,8] and analytical [2,9,10] levels. Furthermore, this current “unfeasibility” can be expanded to the handling of the data from untargeted metabolomics studies: how do we maximize the value of untargeted metabolomic data [11] with the current chemometric methods and algorithms?

Metabolomics studies, particularly liquid chromatography mass spectrometry (LC-MS)-based untargeted approaches, generate information-rich, high-dimensional, and complex datasets that remain challenging to handle and fully exploit [2]. Thus, dedicated modeling algorithms, able to cope with the inherent complexity of these metabolomic datasets are mandatory for extracting relevant information. Various chemometric and bioinformatics tools and resources have been developed, and are utilized for this purpose, thereby integrating computer science, mathematics, and statistics [12,13]. However, despite the remarkable progress in the development of tools and algorithms, as presented in the recent review [14], the exhaustive extraction of information from these metabolomic datasets is still a non-trivial undertaking [11].

It is to be noted and emphasized that the information extracted from the metabolomics raw data and the resulting outputs depend heavily on the data analysis methodology employed. Additionally, in the hierarchy of data, information, and knowledge, the logical and epistemological implication is that information is the key to knowledge formulation [12,15,16,17]. In order to maximize the value of metabolomic data and generate biologically-meaningful hypotheses, particularly with regard to the regulatory mechanisms and molecular processes involved in global biological responses (such as those of a biosystem), the metabolomics raw data are to be appropriately handled and fully exploited. This will ensure the extraction of sufficient information to determine, as holistically as possible, biological components that show differential behaviors between experimental conditions [1,11,12,18,19].

Thus, data analysis methodology is critical for generating meaningful scientific results from these information-rich metabolomic data. The typical pipeline used in such analysis has been well detailed and described in the literature, although with some notational and semantic nuances [2,13,17,20,21,22], and can be summarized in the following steps: (i) processing (extracting features from raw instrumental data to a suitable form; normalization, scaling, centering, etc., to put all samples and variables on a comparable scale); (ii) statistical analysis/modeling (this covers understanding and visualization of data and feature selection methods, and validation/estimation of the predictive capability of the applied statistical models); (iii) annotation of the selected features; and (iv) interpretation and metabolic pathway and network analysis, leading to the generation of research hypotheses and knowledge compilation.

The data processing and statistical modeling are crucial and vital, as the information extracted from the raw data will depend on these steps. A better understanding of data processing and statistical algorithms and methods are important to achieve statistically-relevant and optimal biological information. These post-acquisition steps can be challenging and time-consuming and comprise data cleaning and generation of statistical models that are explorative and predictive. Certainly, different parameters and algorithms used in these data analyses steps would lead to different outputs and influence the extent of data mining outcomes [23,24,25]. This observation, thus, raises a series of questions of knowing how to handle untargeted metabolomic data adequately, or if there is one single “formula” or methodological protocol to follow for maximal exploration of untargeted metabolomic datasets.

Considerable literature exists on the data analysis and mathematical description of algorithms and chemometric methods used during data analysis in metabolomics, with suggestions and requirements for a sound approach [14,25,26,27,28,29]. However, questions such as (i) to what extent can an untargeted metabolomic dataset be mined; (ii) are the data analysis methodologies, applied to an untargeted metabolomic dataset, not biased by the scope of the initial biologic question; (iii) are the current chemometric tools and algorithms fit to holistically extract information from the mega-variate datasets generated by untargeted metabolomics studies [30]; (iv) to what extent do the different steps in a data handling methodology pipeline influence the data analysis output; and (v) what could be a methodological approach and practice that would aid to maximize the value of untargeted metabolomic dataset all remain to be explored.

Different from, but also complementing the existing literature on metabolomic data analysis, this study looks at the influence of data pre-processing (e.g., collection parameters, such as intensity threshold and mass tolerance) and pre-treatment methods (e.g., scaling and data transformation) on the statistical models generated and feature selection, using an LC-MS-based untargeted metabolomic dataset. This was in order to actually demonstrate to what extent steps in the data analysis pipeline impact on the output, which would certainly affect the downstream biological interpretation. Thus, the study clearly points out, with illustrative examples, that the methods employed in the data analysis should not be regarded as “one-size-fits-all”. As such, this study intends to make a contribution to the on-going discussions in the metabolomics community with regard to ways of maximizing the value of untargeted metabolomics data and influence/effects of data analysis steps on the downstream analyses and interpretation [11,12,22,31,32]. Ultimately this work emphasizes the importance of understanding the structures of raw data, and exploration of various algorithms and parameters are vital and mandatory in data mining to maximize the value of generated data.

## 2. Results and Discussion

Before embarking on the details of the results, it is firstly worth noting and re-emphasizing that metabolomic data analysis does not occur in isolation, but is rather intimately linked to the other metabolomics workflow steps that are upstream thereof [2,33]. Hence, a careful experimental design is mandatory, and statistical rigor, quality assurance, and proper scientific procedures must be followed and applied at every stage of the workflow so as to generate data that actually contain “objectively true” information about the biological question under investigation [13,28,34,35]. Furthermore, these metabolomics workflow steps are not always to be followed linearly. Adaptation and a “forth-back” methodological approach is necessary to revise, correct (avoid distortions), validate, or to further mine the data so as to obtain a comprehensive biological answer with fewer spurious or false positive outcomes to the research question [13,32].

### 2.1. Data Processing Parameters: Mass Tolerance and Intensity Threshold

Untargeted LC-MS metabolomic analyses generally generate a wealth of data. The data structure is a two-way matrix (retention time and mass spectra directions), with thousands of data entries, depending on the complexity of the extracts, per sample. To make it more complicated, the data inherently contain vast amounts of noise, artifacts, unintended fragments and adducts, potentially making components of the datasets either not usable or redundant [24,36,37]. The challenge is how to extract and create a “clean” dataset from such raw data in a way that captures as much usable information as possible for downstream pattern recognition, classification, and feature selection. Most pre-processing software pipelines share the general functions of peak detection-, alignment, and annotation. Currently, several open-source, as well as commercial software programs, have been developed to aid in metabolomics data processing (up to metabolite annotation for some). Each of these tools has its own capabilities, providing some context-dependent insights, but also limitations [14]. The detailed description of all of these tools and algorithms is beyond the scope of this paper and the reader is referred to the cited literature.

Thus, the first step in metabolomic data analysis is to select relevant signals from the raw data, decrease redundancy and generate a data matrix for downstream analysis. In this study, for creating the data matrix (unbiased mass peak extraction, ion intensities identification and alignment of the acquired LC-MS data), an automated approach was applied, using the MarkerLynx^TM^ Application Manager for MassLynx^TM^ software (Waters Corporation, Manchester, UK) for data processing. As described in the experimental section, the MarkerLynx^TM^ application uses the patented *ApexTrack* peak detection algorithm to perform accurate peak detection and alignment. Following the peak detection, the associated ions are analyzed (the maximum intensity, the Rt and exact *m*/*z* mass) and captured for all samples. The data matrix is then generated [37,38]. The data pre-processing steps and relevant parameters’ settings are detailed in the experimental section.

Varying the mass tolerance parameter (which specifies the mass accuracy of the acquired data) and intensity threshold parameter (which specifies the threshold of a spectral peak) resulted in different data matrices from the same LC-MS raw data, and the different numbers of variables (in the multivariate X-space) and noise levels are tabulated in Table 1. Theoretically, an infinite number of combinations for sets of processing parameters with MarkerLynx^TM^ are possible. In practice, the computational time to process one combination of a set of parameters could be in hours, depending on the size of the datasets. Furthermore, understanding of the underlying algorithms and steps involved in the data processing is essential so as to decide which parameter to vary. As indicated in the experimental section, parameters, such as mass tolerance and the intensity threshold (which define the real peak versus background noise), can be changed, within certain limits: for instance mass tolerance can be set to the mass accuracy of the acquired data (which was 4.9 mDa in this study) and twice this value; hence, in this study mass tolerance was varied in these limits (0.005 and 0.01 Da). The mass tolerance (mass accuracy) parameter is the basis by which the *ApexTrack* algorithm determines the regions of interest in the *m*/*z* domain, whereas the intensity threshold parameter is used in the peak removal step, defining the resultant noise level and redundancy in the data matrix. These two parameters are essential, hence this study explored the impact of these on the creation of the data matrix.

The tabulated results (Table 1), from the same LC-MS raw dataset, demonstrate that changing the mass tolerance and intensity threshold parameters affects the number of defined features (X-variables). One observation to point out is that increasing the intensity threshold (counts) led to a significant decrease in the number of X-variables and noise levels. For a novice in metabolomic data analysis or any metabolomic scientist with less expertise in statistics/chemometrics, this could raise questions with regard to the “correct” method (or set of parameters) to trust and use: e.g., the one producing a matrix with less noise. A point to consider is to what extent any decrease in the number of “defined” ion peaks (variables) would bring about information loss? As a first approach, a chemometrician or statistician would advise for a cleaner data matrix: the less noise in the created matrix the better. Methods (set of parameters or algorithms) that could reduce the noise in the data and decrease the redundancy are often advised and preferred [16,39,40,41,42].

However, the biological insights (the dynamic ranges of metabolite abundance in a biological system, conservation relationships in networks of interrelated compounds, etc.) and the currently inherent analytical limitations (suboptimal quality of extraction, detection and ionization capabilities, etc.) [12,43], call for caution in handling noise elimination and redundant signals in metabolomic datasets. The effort to produce a noise-free and non-redundant data matrix (post-processing) could result in a loss of information, some of which could be informative to comprehensively assess metabolic pathways for a better understanding and description of the regulatory mechanisms underlying the global biological responses [12,18,37,44,45]. Furthermore, as recently demonstrated, some of the ion peaks that could be regarded as a source of redundancy (e.g., adduct formation) might be very crucial and actually needed in metabolite annotation and differentiation [46].

The created matrices (Table 1) were then imported into SIMCA (Soft Independent Modeling of Class Analogy) version 14 software (Umetrics, Umea, Sweden) for statistical modeling: principal component analysis (PCA) and orthogonal projection to latent structures discriminant analysis (OPLS-DA) (generally used approaches in metabolomics data analysis for data overview/descriptive exploration, and explicative/predictive analysis, respectively). Total variation in metabolomic data is multifactorial, comprising of the sum of biological variation (induced and non-induced) and technical variation [2,27,47]. Therefore such data, with its inherent properties and structures (including large number of variables, nonlinearity, heteroscedasticity, missing values), imperatively requires special attention during statistical handling to avoid a risk of model overfitting [48], manipulation, and confusion of statistical findings and distortion of the results, which can lead to incorrect data interpretation and false discovery [32,49].

Before performing PCA and OPLS-DA, the data was mean-centered (to put all variables on equal footing) and Pareto-scaled (to adjust for measurement errors as to have homoscedasticity in the data). There are different methods for dealing with missing values, and each method/approach impacts on downstream statistical analyses [31,50,51]. In the present study, the SIMCA software uses an adjusted nonlinear iterative partial least squares (NIPALS) algorithm (with a correction factor of 3.0) [52] in handling the missing values. The threshold of missing values is, by default, 50%, and in the four matrices (from the same raw data, Table 1) no observations or variables had missing values exceeding the permitted tolerance. Thus, to assess the effect of processing parameters (mass tolerance and intensity threshold, Table 1) on the statistical output, the quality and characteristics of computed PCA and OPLS-DA models were comparatively evaluated. The statistically-extracted discriminating variables from the four scenarios (Table 1) were also compared.

**Figure 1:**
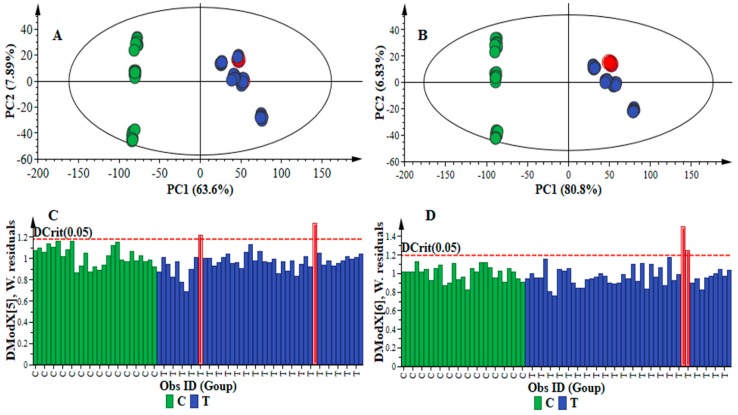
PCA score scatterplots and distance to the model (DModX) plot. (**A**) Score scatterplot of the PCA model of data X (processed with *Method 1:* Table 1): a five-component model, explaining 78.6% variation in the Pareto-scaled data and the amount of predicted variation by the model, according to cross-validation, is 74.6%; (**B**) Score scatterplot of the PCA model of data X (processed with *Method 4*: Table 1): a 6-component model, explaining 93.4% variation in the Pareto-scaled data X and the amount of predicted variation by the model, according to cross-validation, is 91.7%; (**C**) The DModX plot of the PCA model in (**A**) showing the moderate outliers (in red); and (**D**) The DModX plot of the PCA model in (**B**) showing the moderate outliers (in red).

For principal component (PC) analyses, the results showed that varying the processing parameters (mass tolerance and intensity threshold) affected the maximum (suitable) number of computed PCs, optimized using seven-fold cross-validation, to explain the variation in data X: changing from a five-PCs to a six-PCs models (Table 2). Notably, only the “*R*1” significant components, i.e., those producing an increase in Q^2^, were retained. Although, visually, the sample clustering in the PCA scores space (constructed from the first two PCs) show no significant difference across the four datasets (Figure 1A,B and Figure S1A,B), the model quality was clearly affected. This can be assessed by inspecting the PCA parameters and diagnostic tools, which are computed and displayed graphically or numerically. In computing a PC model, strong and moderate outliers (observations that are extreme or do not fit the model) are often formed. Strong outliers have a high leverage on the model, shifting it significantly and reducing the predictability, whereas the moderate outliers correspond to the temporary perturbations (in the process/study), indicating a shift in the process/study behavior [53,54].

Strong outliers are identified from scores and Hotelling’s T^2^ range plots. The latter is a multivariate generalization of Student’s *t*-test, providing a check for observation adhering to multivariate normality [53]. When used in conjunction with a scores plot, the Hotelling’s T^2^ defines the normality area corresponding to 95% confidence in this study. Inspecting the scores and Hotelling’s T^2^ range plots for the calculated four PC models (Figure 1A,B and Figure S2), no strong outliers were observed. The moderate outliers, on the other hand, are identified by inspecting the model residuals (X-variation that was not captured by the PC model). The detection tool for the moderate outliers is the distance to the model in X-space (DModX), with a maximum tolerable distance (Dcrit) [53].

In this study, for all four datasets (Table 1), the DModX was normalized in units of standard deviation, with the significance level of 0.05. Inspecting the DModX plots (Figure 1C,D and Figure S1C,D) showed the existence of some moderate outliers. What is important to notice is that these moderate outliers were different in the four PC models (Figure 1C,D and Figure S1C,D), suggesting that varying of the two processing parameters (mass tolerance and intensity threshold) clearly altered the structure in the X-space (particularly in higher-order components), and this impacts the statistical description thereafter. The moderate outliers were further investigated by computing the contribution plots (Figure S3) and no sample/observation had variable(s) with critical deviation from the rest of the dataset.

Furthermore, the model fit (R^2^X) and predictive power (Q^2^) diagnostic parameters were evaluated for the computed four PC models. The model fit informs how well the data of the training set can be mathematically reproduced indicating, quantitatively, the goodness of fit for the computed model. The R^2^X, thus, quantitatively describes the explained variation in the modeled X-space [25,55]. The predictive ability of the model, on the other hand, was estimated using cross-validation, providing a quantitative measure of the predicted variation in X-space. A change in data processing parameters (mass tolerance and intensity threshold) clearly affected PCA, altering the model quality. The positive change in both mass tolerance and intensity threshold parameters resulted in an increase in R^2^X and Q^2^, with a substantial difference observed in the predicted variation, Q^2^ (Table 2). These results demonstrate that the upstream metabolomic data processing and treatment affect the outcome of the statistical analyses, which then would impact, both quantitatively and qualitatively, the mining of “what the data says” [49].

**Figure 2:**
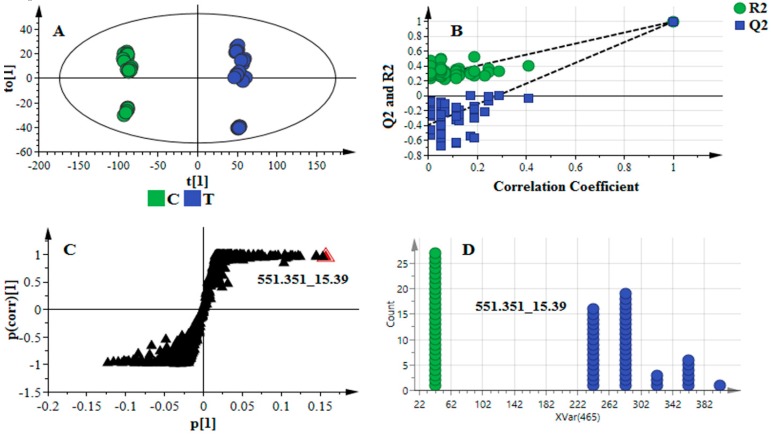
OPLS-DA model for data X (processed with *Method 4*, Table 1). The labels C and T refer to control (green) and treated (blue), respectively. (**A**) A score plot showing group separation in an OPLS-DA score space; (**B**) the response permutation test plot (*n* = 50) for the OPLS-DA model in (**A**): the R^2^ and Q^2^ values of the permutated model are represented on the left-hand side of the plot, corresponding to *y*-axis intercepts (Table 2): R^2^ = (0.0, 0.271) and Q^2^ = (0.0, −0.340); (**C**) an OPLS-DA loading S-plot for the “*Method 4*” model. The *x*-axis is the modelled covariation and the *y*-axis is the loading vector of the predictive component (modeled correlation). Variables situated far out in the S-plot are statistically relevant and represent possible discriminating variables; and (**D**) the dot plot of the selected (marked) variable in S-plot (**C**), showing that such a variable is a very strong discriminating variable, as it has no overlap between groups.

For supervised multivariate analyses (OPLS-DA in this case), the calculated models were validated and assessed. Firstly, to note that for all the OPLS-DA models of the four datasets, there was clear discrimination between the sample groups in the scores space (Figure 2A and Figure S4). The analysis of variance testing of cross-validated predictive residuals (CV-ANOVA) was used to assess the reliability of the obtained models [56]. The computed OPLS-DA models for the four datasets, to separate multivariate relationships into predictive and orthogonal variation, were statistically good models with *p*-values significantly lower than 0.05 (Table 2). Furthermore, the response permutation test (with *n* = 50) was used to validate the predictive capability of the computed OPLS-DA models. In this statistical test the R^2^ and Q^2^ values of the true model are compared with that of the permutated model. The test is carried out by randomly assigning to the two different groups, after which the OPLS-DA models are fitted to each permutated class variable. The R^2^ and Q^2^ values are then computed for the permutated models and compared to the values of the true models [57,58].

The results indicate that the calculated models have much higher R^2^ and Q^2^ values (Figure 2B and Table 2) and, thus, the computed true OPLS-DA models are statistically far better than the 50 permutated models for each dataset. Assessing the total variation in X-space (predictive and orthogonal) explained by the models, the results show that the R^2^X values were different: a change in mass tolerance and intensity threshold affect the amount of variation explained by the computed models (Table 2). For variable selection, the OPLS-DA loading S-plots were evaluated (Figure 2C). This loading plot has an S-shape provided the data are centered/Pareto-scaled, and aids in identifying variables which differ between groups (discriminating variables), i.e., variables situated at the upper right or lower left sections in the S-plot. The *p*_1_-axis describes the influence of each X-variable on the group separation (modeled covariation), and the *p*(corr)_1_-axis represents the reliability of each X-variable for accomplishing the group separation (modeled correlation). Variables that combine high model influence (high covariation/magnitude) with high reliability (i.e., smaller risk for spurious correlation) are statistically relevant as possible discriminating variables [25,59]: |*p*[1]| ≥ 0.05 and |*p*(corr)| ≥ 0.5 in this study.

Furthermore, to avoid variable selection bias [60,61], the significance of the variables from the loading S-plot was assessed using, firstly, the variable importance in projection (VIP) plot (SIMCA 14 software). The latter summarizes the importance of the variables both to explain X and to correlate to Y. The higher the VIP value (exceeding 1.0) the more significant is the variable in the complex analysis in comparing the difference between two or more groups [62,63]. Each selected variable from the S-plot (with high model influence and reliability, and VIP score >> 1.0) was further evaluated using a dot plot (Figure 2D). The latter is similar to a histogram and kernel density estimation (but algorithmically different), computing each observation as a unit: the observations are sorted into “bins” representing variable sub-ranges [64]. A very strong discriminating variable has no overlap between groups (Figure 2D). The mathematical description of the mentioned algorithms and methods (e.g., VIP, dot plot, etc.) is beyond the scope of this paper; the reader is, thus, referred to the cited literature.

**Figure 3:**
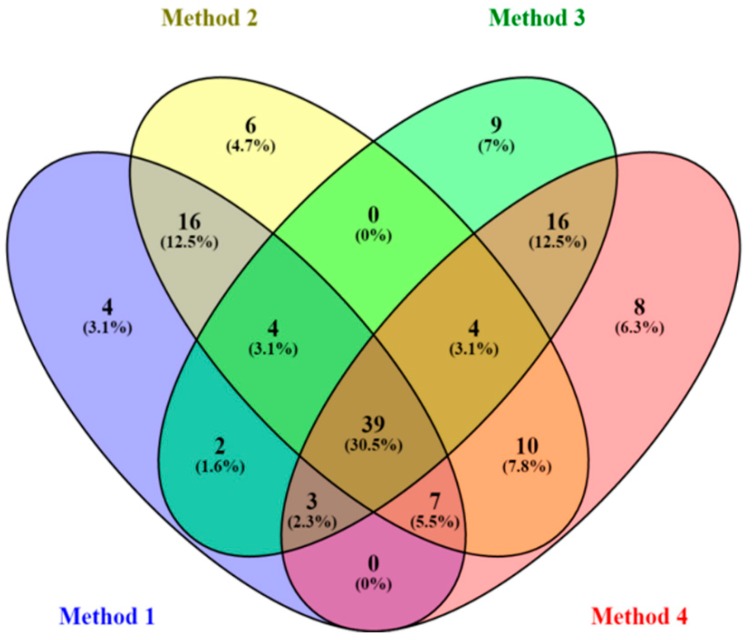
Venn diagram displaying (comparatively) the statistically-selected discriminating variables from the four OPLS-DA models (of the four different pre-processing methods, Table 1 and Table 2). The four pre-processing methods, applied on the same raw data, generated four different data matrices; and the statistical analyses of the four matrices led to different discriminating variables (with some overlap), as graphically depicted in the diagram.

The statistically-validated discriminating variables from each model (representing each dataset generated from varying the two mentioned processing parameters) were then compared. The results demonstrate that the change in pre-processing parameters (mass tolerance and intensity threshold, in this case) affected the downstream statistical analyses, particularly the statistically-selected variables: comparing these variables showed some overlap, but also each method had unique variables (Figure 3). This observation compliments and corroborates the above PCA results that data processing and treatment (prior to statistical analyses) alter not only the infographics, but also the extracted information, which might impact the interpretation thereafter.

### 2.2. Data Scaling and Transformation Influence

To also evaluate the effect of data (pre)-treatment algorithms on downstream chemometric models, different scaling and transformation methods were applied on the data matrix created using *Method 1* (Section 3.1, Table 1). The scaling methods explored are center (Ctr), autoscaling (also termed unit variance (UV)), and Pareto, and the transformation methods used were logarithmic and power transformation (the formulae are described in the experimental section). To avoid confusion, the definitions for scaling and transformation methods used are those in the SIMCA manual (User’s Guide to SIMCA 13, 2012), which are also related to descriptions found in the cited literature [27,65].

Following scaling and transformation, PCA and OPLS-DA models were constructed/fitted and evaluated to assess the influence of these data pre-treatment methods on the models’ quality, classification accuracy, feature selection/extraction, and the subsequent interpretability of the data. The data pre-treatment is an essential step in the metabolomic data analysis pipeline as it enables the preparation of the data for downstream analyses, minimizing variable redundancy and making all variables more comparable in size [22,31,65].

**Figure 4:**
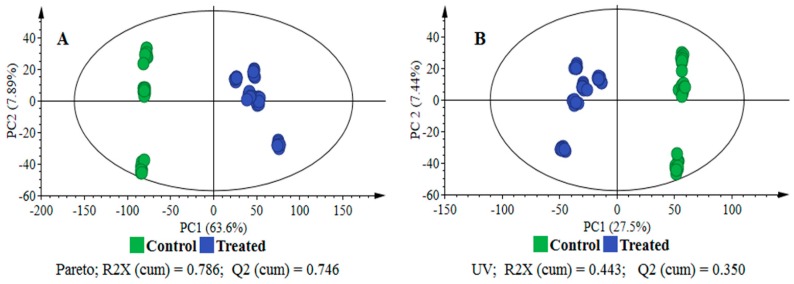
PCA score scatterplots. PCA models of the same data X, but with different scaling methods. (**A**) A five-component model, explaining 78.6% variation in the Pareto-scaled data, X, and the amount of predicted variation by the model, according to cross-validation, is 74.6%; and (**B**) A five-component model, explaining 44.3% variation in the unit variance (UV)-scaled data, X, and the amount of predicted variation by the model, according to cross-validation, is 35.0%.

For PC analyses, the results showed that scaling and transformation methods affected the dimension of the PC-space optimized using seven-fold cross-validation, the sample clustering in the PCA scores space (e.g., constructed from the first two PCs), and the moderate outliers detected in the DModX plots (Figure 4 and Figure S5). Furthermore, the metric used to assess the model fit (or explained variation) and predictive ability of the computed PCA models were R^2^ and Q^2^ [2,25,55]. The inspection of these diagnostic metrics shows that scaling and/or transformation remarkably affected the amount of explained variation (the goodness of fit) by the model and its predictive ability (Table 3).

**Figure 5:**
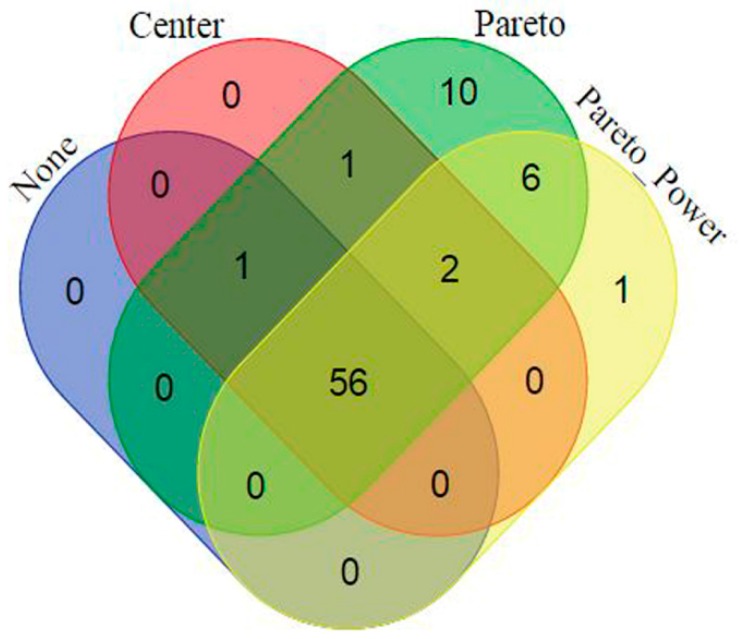
Venn diagram displaying (comparatively) the statistically-selected discriminating variables from the four OPLS-DA models that are statistically valid (Table 3). As indicated in the diagram, there are unique and shared discriminating variables from the four models i.e., different data pre-treatment (scaling and transformation) methods led to different discriminating variables.

As in Section 2.1, for supervised multivariate analyses (OPLS-DA in this case), the calculated models were validated, and the robustness and reliability of the models assessed. In addition to the R^2^ and Q^2^ metrics, the CV-ANOVA was used to assess the reliability of the obtained models [56] and the response permutation test (with *n* = 50) was used to validate the predictive capability of the computed OPLS-DA models [57,58]. Furthermore, in both Section 2.1 and Section 2.2, predictive testing was also employed to assess the best pre-processing and pre-treatment workflow (Figures S6 and S7). The results tabulated in Table 3 demonstrate that the scaling and transformation methods affected significantly not only the explained variation R^2^ (both predictive and orthogonal) but also the classification accuracy, reliability, predictive capability of the model and, subsequently, extracted variables (Figure 5). The supervised learning models computed following for instance UV-scaling and/or log-transformation (particularly in this case), would not be chemometrically/statistically trusted as the classification of these models could be by chance, as indicated by the permutation validation tests (lower R^2^ values compared to the permutated models, Table 3).

These results (Table 3) demonstrate that the choice of pre-treatment method(s) is crucial and may depend not only on the biological information to be acquired but also on the data analysis method to follow. For instance, a chemometric approach/method that focuses on (dis)similarities would differ from the PCA attempting to explain as much variation as possible in as few components as possible. Thus, varying data properties using data pre-treatment methods may, for instance, enhance the results of a clustering method, while obscuring the results of a PCA model [22,27].

These results (varying pre-processing parameters and pre-treatment methods) clearly demonstrate that handling the multivariate data from untargeted metabolomic analyses is indeed challenging. Both Figure 3 and Figure 5 depict that the data analysis outcome of an untargeted metabolomic data is remarkably influenced by the upstream data handling, such as pre-processing and pre-treatment methods, and the algorithms applied. The symbiosis of chemometrics and metabolomics [66] is illustrated here, with a clear demonstration that an understanding of data structures and data analysis methodologies is mandatory for stepping forward from data to information as comprehensively as possible.

Furthermore, Figure 3 and Figure 5 then raise questions with regard to what could be the best methodological approach for data pre-processing and pre-treatment, given an untargeted metabolomics dataset (as it is in this case) that exhaustively explores the data. Could these results (including the tabulated models’ description in Table 2 and Table 3) actually be pointing to the problem of “multiplicity of good models”/“multitude of descriptions” (Rashomon Effect) [67]? Or is it, indeed, an indication that, currently, no single chemometric method can actually extract all of the information from an untargeted metabolomic dataset. Different chemometric methodologies and algorithms are constantly being developed to cope with systems biology-perspective demands [30], but maybe the chemometric principle of “largest variance is most important” might not hold true in all cases, as the total variation in the metabolomic data is multifactorial. On the other hand, to extract relevant information may require searching the “needle in the haystack” methods [12,30,47].

There is certainly a “symphony” of data analysis strategies and approaches throughout the pipeline, from post-acquisition steps to the variable selection. Algorithms or methods used at each step of the pipeline affect the downstream analyses and outcome [31,33,49]. In this study, using the same LC-MS raw data, but changing the pre-processing parameters (Section 3.1) and data pre-treatment methods (Section 3.2) affected the downstream statistical outcome, thus illustrating (to a certain extent) not only that the metabolomic data are indeed information-rich, but also the limitation in existing chemometric methods and the need of uttermost care in data handling. Furthermore, the results demonstrate that, currently, the possible “Archimedean” methodical point for an optimal extraction of information from untargeted metabolomic datasets could be the exploration of existing chemometric and statistical methods at each step of data analysis pipeline.

**Figure 6:**
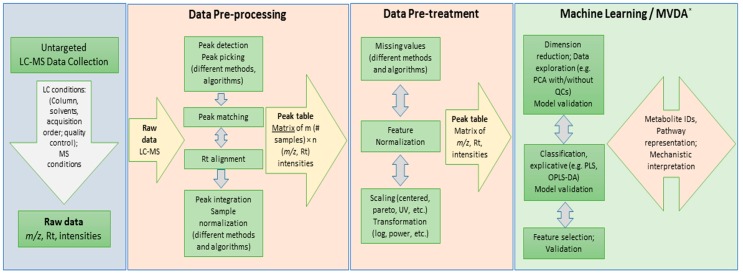
Flowchart displaying an overview of a typical LC-MS data mining pipeline. Different post-acquisition steps involved in data analysis: data pre-processing and pre-treatment (focus of this study) and machine learning/multivariate data analysis (MVDA). Each step consists of a typical workflow to follow and there are different methods and algorithms that can be employed.

However, these observations and generalizations are built on an assumption that the study design and data quality assurance are always correctly executed and considered [35,42,68], and the underlying philosophy of chemometrics is efficiently applied throughout the metabolomics study from the start to biological interpretation [12,25,30]. To maximize the value of metabolomic data, exploration of different algorithms and methods might be the best trade-off currently. As this study demonstrates, different steps involved in data mining are interdependent (Figure 6), and methods employed in each step would influence the downstream steps. However, although an exploration of different algorithms and methods is encouraged, this should be guided and discerned based on a thorough manual examination of the raw data and a strong analytical and chemometric knowledge.

## 3. Materials and Methods

### 3.1. Dataset and Raw Data Processing

Raw data from an untargeted ultra-high performance liquid chromatography-mass spectrometry (UHPLC-MS) metabolomics study, in this case of sorghum plants responding dynamically to infection by a fungal pathogen, *Colletotrichum sublineolum*, was employed. Briefly, two groups of samples were used in this study: fungal-infected (treated) and control (non-infected) samples, labelled T and C, respectively. The control group consisted of nine samples, whereas the treated group consisted of 15 samples. Each sample was a methanol extract from 10 plants. Analytical data of methanol-based plant extracts were acquired in both positive and negative centroid ion mode; but for this paper, only the positive data were further processed. The *m*/*z* range was 100–1000 Da and the data were acquired by applying a Waters Acquity UHPLC system coupled in tandem to a Waters photodiode array detector and an electrospray SYNAPT G1 HDMS Q-TOF mass spectrometer (Waters, Milford, MA, USA), applying a method as previously described [69]. A lock spray source was used allowing online mass correction to obtain high mass accuracy of analytes. Leucine encephalin, [M + H]^+^ = 556.2766 and [M − H]^−^ = 554.2615, was used as the lock mass, being continuously sampled every 15 s, thus producing an average intensity of 350 counts scan^−1^ in centroid mode. By using a lock mass spray as a reference and continuously switching between sample and reference, the MassLynx^TM^ software can automatically correct the centroid mass values in the sample for small deviations from the exact mass measurement.

Quality control (QC) pooled samples were used to condition the LC-MS analytical system so as to assess the reliability and reproducibility of the analysis, and for non-linear signal correction [42,70,71,72]. Sample acquisition was randomized and the QC sample (six injections) was analyzed every 10 injections to monitor and correct changes in the instrument response, with each sample being injected three times. Furthermore, six QC runs were performed at the beginning and end of the batch to ensure system equilibration. Such sample randomization provides stochastic stratification in sample acquisition so as to minimize measurement bias. In the PCA space, the QC samples were clustered closely to each other (results not shown, as it is not the focus of this study), thus confirming the stability of the LC-MS system used, the reliability, and reproducibility of the analysis.

### 3.2. Dataset Matrix Creation and Data Pre-Treatment

Visualization and data processing were performed using MassLynx XS^TM^ 4.1 software (Waters Corporation, Manchester, UK). Only the centroid electrospray ionization (ESI) positive raw data were used in this study. The MarkerLynx^TM^ application manager of the MassLynx software was used for data pre-processing (matrix creation). Four dataset matrices (hereafter referred to as *Methods*) were created by changing mass tolerance and intensity threshold settings: *Method 1* (mass tolerance of 0.005 Da and intensity threshold of 10 counts), *Method 2* (mass tolerance of 0.005 Da and intensity threshold of 100 counts), *Method 3* (mass tolerance of 0.01 Da and intensity threshold of 10 counts), and *Method 4* (mass tolerance of 0.01 Da and intensity threshold of 100 counts). For all of the *Methods*, the parameters of the MarkerLynx^TM^ application were set to analyze the 1–15 min retention time (Rt) range of the mass chromatogram, mass range 100–1000 Da, and alignment of peaks across samples within the range of ±0.05 Da and ±0.20 min mass and Rt windows, respectively.

The MarkerLynx^TM^ application uses the patented *ApexTrack* (termed also *ApexPeakTrack*) algorithm to perform accurate peak detection and alignment. MarkerLynx^TM^ initially determines the regions of interest in the *m*/*z* domain based on mass accuracy (mass tolerance). The *ApexTrack* algorithm controls peak detection by peak width (peak width at 5% height) and baseline threshold (peak-to-peak baseline ratio) parameters. In this study, these parameters were calculated automatically by MarkerLynx^TM^. The *ApexTrack* also calculates the baseline noise level using the slope of inflection points. Thus, for peak detection, the *ApexTrack* algorithm consists of taking the second derivative of a chromatogram and locates the inflection points, the local minima, and peak apex for each peak, to decide the peak area and height. A “corrected” Rt is then assigned and the data are correctly aligned, with the alignment of peaks across samples within the range of user-defined mass and Rt windows. Following the peak detection, the associated ions are analyzed (the maximum intensity, its Rt and exact *m*/*z* mass) and captured for all samples.

An additional data cleaning step, a peak removal step denoted by user-defined peak intensity threshold (and noise elimination level) parameter, is conjugated to the alignment algorithm: briefly, if a peak is above threshold in one sample and if it is lower than the threshold in another sample it lowers the threshold for that sample until it reaches the noise elimination level. The noise is understood as residual peaks in the background (from electronics, nebulizer gas, solvents, cleanliness of source, column, etc.) and/or below the noise elimination threshold. MarkerLynx^TM^ also performs data normalization. In this study normalization was done by using total ion intensities of each defined peak. Prior to calculating intensities, the software performs a patented modified Savitzky-Golay smoothing and integration.

Although parameters, such as mass tolerance and intensity threshold (which define the real peak versus noise), can be regarded as relatively instrument-dependent (or a property of acquired data), they can be changed (within certain limits): mass tolerance can be set to the mass accuracy of the acquired data (which was 4.9 mDa in this study) and twice this value; hence, in this study mass tolerance was varied within these limits (0.005 and 0.01 Da). Considering the complexity and high-dimensionality of the samples (particularly in plant metabolomics), and considering the mathematical limitations of chemometric algorithms, it is essential to explore the processing methods (combinations of sets of parameters: selected as objectively and optimally as possible) so as to maximize the mining of the raw data.

The MarkerLynx^TM^-generated data matrices were exported into SIMCA software, version 14 (Umetrics, Umea, Sweden) for statistical analyses. An unsupervised method, principal component analysis (PCA), and a supervised modeling, orthogonal projection to latent structures-discriminant analysis (OPLS-DA), were employed. The data pre-treatment methods used included scaling and transformation. These two types of data pre-treatment were explored as described in Section 2.2. The scaling methods looked at were center (Ctr), autoscaling, (also known as unit variance, UV) and Pareto, and the transformation methods used were logarithmic and power transformation. The formulae (or mathematical description of these methods) can be found in the cited literature [27] and in the SIMCA version 13 manual (User’s Guide to SIMCA 13, 2012). In this study, the logarithmic transformation was 10Log (C1 × X + C2) where C1 = 1 and C2 = 0; and the power transformation was (C1 × X + C2) ^C3^ where C1 = 1, C2 = 0, and C3 = 2. As described in the results, the computed models were validated.

## 4. Conclusions and Perspectives

Using the same raw dataset, and exploring and applying different methods and algorithms in handling the data, the study clearly demonstrates how crucial the data pre-processing and pre-treatment steps are in a metabolomics data analysis pipeline. These steps significantly affect the chemometric models computed downstream, including the variation explained by the models, the classification accuracy and the quality of the models. However, the inferred observations from this study are limited, as being drawn from a single dataset (from plant samples). Applying the same exercise to different datasets from other sample types (e.g., cell extracts, biofluids, etc.) might provide more insights and subsequently a formulation of generalized guidelines. Thus, it suffices here to point out that an understanding of the data structures and the approach adopted for handling a specific untargeted metabolomic dataset will definitely influence the data analysis outcome, as demonstrated in this study. Stepping forward from untargeted metabolomic data, to information, and finally knowledge, is not a trivial endeavor or a simple “syllogistic” approach.

As encouraged by the Metabolomics Society [21,28,73,74], a proper and detailed reporting of the data analysis methodology used in a metabolomic study is essential and “ethically” obligatory, for clarity of the etiology of the inferences of the study and ascertaining the reproducibility of the latter. Furthermore, the growing call for submission of metabolomic studies (and raw data) into the repositories, such as MetaboLights [75,76,77], is to be encouraged, as further mining of datasets (with different data analysis scopes) can yield more information and more biological insights [11,22]. Untargeted metabolomic studies, in general, generate large amounts of data that are exceedingly rich in information and, consequently, realistically challenging to mine, interpret, and pursue mechanistically in a comprehensive biological context. Furthermore, even though the current study used only one method for peak detection (and varying its parameters), it should be noted that there are a wide variety of workflows (vendor-specific, commercial software, and freeware) available for peak picking/detection. Since various algorithms are used by these different peak detection methods, the choice thereof can have a significant influence on the processed end results of a study [14,33,78,79,80]. Hence, careful and thoughtful use of pre-processing and processing tools is mandatory to be able to make a critical judgment on the outcomes following those two essential steps in the metabolomics workflows. Furthermore, exploration of data analysis methods (as demonstrated in this study) and data sharing (via data repositories) are encouraged so as to maximize the value of these metabolomic datasets.

## Figures and Tables

**Table 1:** Parameters associated with the different datasets generated from MarkerLynx^TM^ processing (Section 3.2).

Data Set	Mass Tolerance (Da)	Intensity Threshold (counts)	X-Variable	Noise Level (%)
***Method 1***	0.005	10	6989	24
***Method 2***	0.005	100	720	9
***Method 3***	0.01	10	7309	23
***Method 4***	0.01	100	765	8

**Table 2:** Generated PCA and OPLS-DA models of the four dataset matrices described as *Methods* *1–4* (Section 3.2).

Data Set	Model Quality and Description								
	PCA			OPLS-DA					
	#PC	R^2^X (cum)	Q^2^ (cum)	R^2^X (cum)	R^2^Y (cum)	Q^2^ (cum)	CV-ANOVA *p*-Value	Permutation (*n* = 50)	
								R^2^	Q^2^
***Method 1***	5	0.786	0.746	0.740	0.997	0.995	0.000	(0.0, 0.573)	(0.0, −0.330)
***Method 2***	5	0.926	0.902	0.857	0.988	0.987	0.000	(0.0, 0.0552)	(0.0, −0.212)
***Method 3***	6	0.793	0.744	0.689	0.989	0.986	0.000	(0.0, 0.304)	(0.0, −0.358)
***Method 4***	6	0.934	0.917	0.894	0.997	0.997	0.000	(0.0, 0.271)	(0.0, −0.340)

**Table 3:** Statistics of computed PCA and OPLS-DA models illustrating the effect of scaling and transformation on the dataset matrix for *Method 1.*

Data Treatment		Model Quality and Description							
		PCA		OPLS-DA					
Scaling	Trans-Formation	R^2^X (cum)	Q^2^ (cum)	R^2^X (cum)	R^2^Y (cum)	Q^2^ (cum)	CV-ANOVA *p*-Value	Permutation (*n* = 50)	
								R^2^	Q^2^
None	**None**	0.995	0.981	0.981	0.852	0.849	5.34 × 10^−23^	(0.0, 0.128)	(0.0, −0.213)
Center	**None**	0.959	0.923	0.923	0.991	0.988	0.000	(0.0, 0.161)	(0.0, −0.329)
UV	**None**	0.443	0.350	0.337	0.992	0.986	0.000	(0.0, 0.650)	(0.0, −0.294)
Pareto	**None**	0.786	0.746	0.740	0.997	0.995	0.000	(0.0, 0.573)	(0.0, −0.330)
UV	**Log**	0.641	0.517	0.548	0.998	0.996	0.000	(0.0, 0.665)	(0.0, −0.222)
Pareto	**Log**	0.667	0.517	0.548	0.998	0.996	0.000	(0.0, 0.633)	(0.0, −0.184)
UV	**Power**	0.435	0.336	0.307	0.994	0.988	0.000	(0.0, 0.649)	(0.0, −0.311)
Pareto	**Power**	0.948	0.900	0.922	0.993	0.990	0.000	(0.0, 0.267)	(0.0, −0.480)

UV = Unit variance scaling.

## Supplementary Materials

The following are available online at http://www.mdpi.com/2218-1989/6/4/40/s1, Figure S1: PCA scores and DModX plots (of *Methods 2* and *3* in Table 2), Figure S2: Hotelling’s T^2^ range plots of the four PCA models (*Methods 1* to *4* in Table 2), Figure S3: DModX and a typical contribution plots (of PCA models for the *Method 1* data set), Figure S4: OPLS-DA scores plots, Figure S5: DModX plots for the detection of moderate outliers, Figure S6: Predicted scores plots and DModXPS, Figure S7: The Coomans’ plots—distance to model predicted (DModXPS+) of two models.

## References

[1] Sévin D.C., Kuehne A., Zamboni N., Sauer U. (2015). Biological insights through nontargeted metabolomics. Curr. Opin. Biotechnol..

[2] Tugizimana F., Piater L.A., Dubery I.A. (2013). Plant metabolomics: A new frontier in phytochemical analysis. S. Afr. J. Sci..

[3] Okazaki Y., Saito K. (2012). Recent advances of metabolomics in plant biotechnology. Plant Biotechnol. Rep..

[4] Bartel J., Krumsiek J., Theis F.J. (2013). Statistical methods for the analysis of high-throughput metabolomics data. Comput. Struct. Biotechnol. J..

[5] Worley B., Powers R. (2013). Multivariate analysis in metabolomics. Curr. Metabol..

[6] Choi Y.H., Verpoorte R. (2014). Metabolomics: What you see is what you extract. Phytochem. Anal..

[7] Duportet X., Aggio R.B.M., Carneiro S., Villas-Bôas S.G. (2012). The biological interpretation of metabolomic data can be misled by the extraction method used. Metabolomics.

[8] Yanes O., Tautenhahn R., Patti G.J., Siuzdak G. (2011). Expanding coverage of the metabolome for global metabolite profiling. Anal. Chem..

[9] Sumner L.W., Mendes P., Dixon R.A. (2003). Plant metabolomics: Large-scale phytochemistry in the functional genomics era. Phytochemistry.

[10] Allwood J.W., Ellis D.I., Goodacre R. (2008). Metabolomic technologies and their application to the study of plants and plant-host interactions. Physiol. Plant..

[11] Goeddel L.C., Patti G.J. (2012). Maximizing the value of metabolomic data. Bioanalysis.

[12] Boccard J., Rudaz S. (2014). Harnessing the complexity of metabolomic data with chemometrics. J. Chemom..

[13] Beisken S., Eiden M., Salek R.M. (2015). Getting the right answers: Understanding metabolomics challenges. Expert Rev. Mol. Diagn..

[14] Misra B.B., van der Hooft J.J.J. (2016). Updates in metabolomics tools and resources: 2014–2015. Electrophoresis.

[15] Kell D.B., Oliver S.G. (2004). Here is the evidence, now what is the hypothesis? The complementary roles of inductive and hypothesis-driven science in the post-genomic era. BioEssays.

[16] Boccard J., Veuthey J.-L., Rudaz S. (2010). Knowledge discovery in metabolomics: An overview of MS data handling. J. Sep. Sci..

[17] Goodacre R., Vaidyanathan S., Dunn W.B., Harrigan G.G., Kell D.B. (2004). Metabolomics by numbers: Acquiring and understanding global metabolite data. Trends Biotechnol..

[18] Cicek A.E., Roeder K., Ozsoyoglu G. (2014). MIRA: Mutual information-based reporter algorithm for metabolic networks. Bioinformatics.

[19] Toubiana D., Fernie A.R., Nikoloski Z., Fait A. (2013). Network analysis: Tackling complex data to study plant metabolism. Trends Biotechnol..

[20] Brown M., Dunn W.B., Ellis D.I., Goodacre R., Handl J., Knowles J.D., O’Hagan S., Spasić I., Kell D.B. (2005). A metabolome pipeline: From concept to data to knowledge. Metabolomics.

[21] Sumner L.W., Amberg A., Barrett D., Beale M.H., Beger R., Daykin C.A., Fan T.W.-M., Fiehn O., Goodacre R., Griffin J.L. (2007). Proposed minimum reporting standards for chemical analysis. Metabolomics.

[22] Gromski P.S., Xu Y., Hollywood K.A., Turner M.L., Goodacre R. (2015). The influence of scaling metabolomics data on model classification accuracy. Metabolomics.

[23] Yang J., Zhao X., Lu X., Lin X., Xu G. (2015). A data preprocessing strategy for metabolomics to reduce the mask effect in data analysis. Front. Mol. Biosci..

[24] Boccard J., Rudaz S. (2013). Mass spectrometry metabolomic data handling for biomarker discovery. Proteomic and Metabolomic Approaches to Biomarker Discovery.

[25] Trygg J., Holmes E., Lundstedt T. (2007). Chemometrics in Metabonomics. J. Proteome Res..

[26] De Livera A.M., Sysi-Aho M., Jacob L., Gagnon-Bartsch J.A., Castillo S., Simpson J.A., Speed T.P. (2015). Statistical methods for handling unwanted variation in metabolomics data. Anal. Chem..

[27] Van den Berg R.A., Hoefsloot H.C.J., Westerhuis J.A., Smilde A.K., Werf M.J. (2006). Van Der Centering, scaling, and transformations: Improving the biological information content of metabolomics data. BMC Genom..

[28] Goodacre R., Broadhurst D., Smilde A.K., Kristal B.S., Baker J.D., Beger R., Bessant C., Connor S., Capuani G., Craig A. (2007). Proposed minimum reporting standards for data analysis in metabolomics. Metabolomics.

[29] Saccenti E., Hoefsloot H.C.J., Smilde A.K., Westerhuis J.A., Hendriks M.M.W.B. (2013). Reflections on univariate and multivariate analysis of metabolomics data. Metabolomics.

[30] Buydens L. (2013). Towards tsunami-resistant chemometrics. Anal. Sci..

[31] Di Guida R., Engel J., Allwood J.W., Weber R.J.M., Jones M.R., Sommer U., Viant M.R., Dunn W.B. (2016). Non-targeted UHPLC-MS metabolomic data processing methods: A comparative investigation of normalisation, missing value imputation, transformation and scaling. Metabolomics.

[32] Godzien J., Ciborowski M., Angulo S., Barbas C. (2013). From numbers to a biological sense: How the strategy chosen for metabolomics data treatment may affect final results. A practical example based on urine fingerprints obtained by LC-MS. Electrophoresis.

[33] Defernez M., Gall G. (2013). Le strategies for data handling and statistical analysis in metabolomics studies. Advances in Botanical Research.

[34] Moseley H.N.B. (2013). Error analysis and propagation in metabolomics data analysis. Comput. Struct. Biotechnol. J..

[35] Trutschel D., Schmidt S., Grosse I., Neumann S. (2015). Experiment design beyond gut feeling: Statistical tests and power to detect differential metabolites in mass spectrometry data. Metabolomics.

[36] Moco S., Vervoort J., Bino R., Devos R. (2007). Metabolomics technologies and metabolite identification. TrAC Trends Anal. Chem..

[37] Idborg H., Zamani L., Edlund P.-O., Schuppe-Koistinen I., Jacobsson S.P. (2005). Metabolic fingerprinting of rat urine by LC/MS Part 2. Data pretreatment methods for handling of complex data. J. Chromatogr. B.

[38] Stumpf C.L., Goshawk J. (2004). The MarkerLynx application manager: Informatics for mass spectrometric metabonomic discovery. Waters Appl. Note.

[39] Veselkov K.A., Vingara L.K., Masson P., Robinette S.L., Want E., Li J.V., Barton R.H., Boursier-Neyret C., Walther B., Ebbels T.M. (2011). Optimized preprocessing of ultra-performance liquid chromatography/mass spectrometry urinary metabolic profiles for improved information recovery. Anal. Chem..

[40] Cook D.W., Rutan S.C. (2014). Chemometrics for the analysis of chromatographic data in metabolomics investigations. J. Chemom..

[41] Peters S., Van Velzen E., Janssen H.G. (2009). Parameter selection for peak alignment in chromatographic sample profiling: Objective quality indicators and use of control samples. Anal. Bioanal. Chem..

[42] Godzien J., Alonso-Herranz V., Barbas C., Armitage E.G. (2014). Controlling the quality of metabolomics data: New strategies to get the best out of the QC sample. Metabolomics.

[43] Misra B.B., Assmann S.M., Chen S. (2014). Plant single-cell and single-cell-type metabolomics. Trends Plant Sci..

[44] Kohli A., Sreenivasulu N., Lakshmanan P., Kumar P.P. (2013). The phytohormone crosstalk paradigm takes center stage in understanding how plants respond to abiotic stresses. Plant Cell Rep..

[45] Vidal M. (2009). A unifying view of 21st century systems biology. FEBS Lett..

[46] Makola M.M., Steenkamp P.A., Dubery I.A., Kabanda M.M., Madala N.E. (2016). Preferential alkali metal adduct formation by *cis* geometrical isomers of dicaffeoylquinic acids allows for efficient discrimination from their *trans* isomers during ultra-high-performance liquid chromatography/quadrupole time-of-flight mass s. Rapid Commun. Mass Spectrom..

[47] Masson P., Spagou K., Nicholson J.K., Want E.J. (2011). Technical and biological variation in UPLC-MS-based untargeted metabolic profiling of liver extracts: Application in an experimental toxicity study on galactosamine. Anal. Chem..

[48] Hawkins D.M. (2004). The Problem of overfitting. J. Chem. Inf. Comput. Sci..

[49] Broadhurst D.I., Kell D.B. (2006). Statistical strategies for avoiding false discoveries in metabolomics and related experiments. Metabolomics.

[50] Armitage E.G., Godzien J., Alonso-Herranz V., López-Gonzálvez Á., Barbas C. (2015). Missing value imputation strategies for metabolomics data. Electrophoresis.

[51] Ilin A., Raiko T. (2010). Practical approaches to principal component analysis in the presence of missing values. J. Mach. Learn. Res..

[52] Nelson P.R.C., Taylor P.A., MacGregor J.F. (1996). Missing data methods in PCA and PLS: Score calculations with incomplete observations. Chemom. Intell. Lab. Syst..

[53] Wikström C., Albano C., Eriksson L., Fridén H., Johansson E., Nordahl Å., Rännar S., Sandberg M., Kettaneh-Wold N., Wold S. (1998). Multivariate process and quality monitoring applied to an electrolysis process. Part I. Process supervision with multivariate control charts. Chemom. Intell. Lab. Syst..

[54] Eriksson L., Trygg J., Wold S. (2014). A chemometrics toolbox based on projections and latent variables. J. Chemom..

[55] Hawkins D.M., Basak S.C., Mills D. (2003). Assessing model fit by cross-validation. J. Chem. Inf. Comput. Sci..

[56] Eriksson L., Trygg J., Wold S. (2008). CV-ANOVA for significance testing of PLS and OPLS^®^ models. J. Chemom..

[57] Triba M.N., Le Moyec L., Amathieu R., Goossens C., Bouchemal N., Nahon P., Rutledge D.N., Savarin P. (2015). PLS/OPLS models in metabolomics: The impact of permutation of dataset rows on the K-fold cross-validation quality parameters. Mol. BioSyst..

[58] Westerhuis J.A., Hoefsloot H.C.J., Smit S., Vis D.J., Smilde A.K., Velzen E.J.J., Duijnhoven J.P.M., Dorsten F.A. (2008). Assessment of PLSDA cross validation. Metabolomics.

[59] Wiklund S., Johansson E., Sjöström L., Mellerowicz E.J., Edlund U., Shockcor J.P., Gottfries J., Moritz T., Trygg J. (2008). Visualization of GC/TOF-MS-based metabolomics data for identification of biochemically interesting compounds using OPLS class models. Anal. Chem..

[60] Ambroise C., McLachlan G.J. (2002). Selection bias in gene extraction on the basis of microarray gene-expression data. Proc. Natl. Acad. Sci. USA.

[61] Smilde A.K., Westerhuis J.A., Hoefsloot H.C.J., Bijlsma S., Rubingh C.M., Vis D.J., Jellema R.H., Pijl H., Roelfsema F., van der Greef J. (2010). Dynamic metabolomic data analysis: A tutorial review. Metabolomics.

[62] Chong I.-G., Jun C.-H. (2005). Performance of some variable selection methods when multicollinearity is present. Chemom. Intell. Lab. Syst..

[63] Mehmood T., Liland K.H., Snipen L., Sæbø S. (2012). A review of variable selection methods in Partial Least Squares Regression. Chemom. Intell. Lab. Syst..

[64] Wilkinson L. (1999). Dot plots. Am. Stat..

[65] Bro R., Smilde A.K. (2003). Centering and scaling in component analysis. J. Chemom..

[66] Van Der Greef J., Smilde A.K. (2005). Symbiosis of chemometrics and metabolomics: Past, present, and future. J. Chemom..

[67] Breiman L. (2001). Statistical modeling: The two cultures. Stat. Sci..

[68] T’Kindt R., Morreel K., Deforce D., Boerjan W., Bocxlaer J. (2009). Van Joint GC-MS and LC-MS platforms for comprehensive plant metabolomics: Repeatability and sample pre-treatment. J. Chromatogr. B.

[69] Tugizimana F., Steenkamp P.A., Piater L.A., Dubery I.A. (2014). Multi-platform metabolomic analyses of ergosterol-induced dynamic changes in nicotiana tabacum cells. PLoS ONE.

[70] Sangster T., Major H., Plumb R., Wilson A.J., Wilson I.D. (2006). A pragmatic and readily implemented quality control strategy for HPLC-MS and GC-MS-based metabonomic analysis. Analyst.

[71] Sangster T.P., Wingate J.E., Burton L., Teichert F., Wilson I.D. (2007). Investigation of analytical variation in metabonomic analysis using liquid chromatography/mass spectrometry. Rapid Commun. Mass Spectrom..

[72] Dunn W.B., Broadhurst D., Begley P., Zelena E., Francis-McIntyre S., Anderson N., Brown M., Knowles J.D., Halsall A., Haselden J.N. (2011). Procedures for large-scale metabolic profiling of serum and plasma using gas chromatography and liquid chromatography coupled to mass spectrometry. Nat. Protoc..

[73] Jenkins H., Hardy N., Beckmann M., Draper J., Smith A.R., Taylor J., Fiehn O., Goodacre R., Bino R.J., Hall R. (2004). A proposed framework for the description of plant metabolomics experiments and their results. Nat. Biotechnol..

[74] Fiehn O., Sumner L.W., Rhee S.Y., Ward J., Dickerson J., Lange B.M., Lane G., Roessner U., Last R., Nikolau B. (2007). Minimum reporting standards for plant biology context information in metabolomic studies. Metabolomics.

[75] Salek R.M., Haug K., Conesa P., Hastings J., Williams M., Mahendraker T., Maguire E., Gonzalez-Beltran A.N., Rocca-Serra P., Sansone S.-A. (2013). The MetaboLights repository: Curation challenges in metabolomics. Database.

[76] Haug K., Salek R.M., Conesa P., Hastings J., de Matos P., Rijnbeek M., Mahendraker T., Williams M., Neumann S., Rocca-Serra P. (2013). MetaboLights--an open-access general-purpose repository for metabolomics studies and associated meta-data. Nucleic Acids Res..

[77] Rocca-Serra P., Salek R.M., Arita M., Correa E., Dayalan S., Gonzalez-Beltran A., Ebbels T., Goodacre R., Hastings J., Haug K. (2016). Data standards can boost metabolomics research, and if there is a will, there is a way. Metabolomics.

[78] Zhang J., Gonzalez E., Hestilow T., Haskins W., Huang Y. (2009). Review of peak detection algorithms in liquid-chromatography-mass spectrometry. Curr. Genom..

[79] Rafiei A., Sleno L. (2015). Comparison of peak-picking workflows for untargeted liquid chromatography/high-resolution mass spectrometry metabolomics data analysis. Rapid Commun. Mass Spectrom..

[80] Coble J.B., Fraga C.G. (2014). Comparative evaluation of preprocessing freeware on chromatography/mass spectrometry data for signature discovery. J. Chromatogr. A.

