# Supplementary material for: A Conversation on Data Mining Strategies in LC-MS Untargeted Metabolomics: Pre-Processing and Pre-Treatment Steps
Source: Metabolites. 2016 Nov 3;6(4):40. doi: 10.3390/metabo6040040 (PMC5192446; doi:10.3390/metabo6040040)
Supplement: Supplementary file 1 [file metabolites-06-00040-s001.pdf]

metabolites

Supplementary Materials: A Conversation on Data Mining Strategies in LC-MS Untargeted Metabolomics: Pre-Processing and Pre-Treatment Steps

Fidele Tugizimana, Paul A. Steenkamp, Lizelle A. Piater and Ian A. Dubery

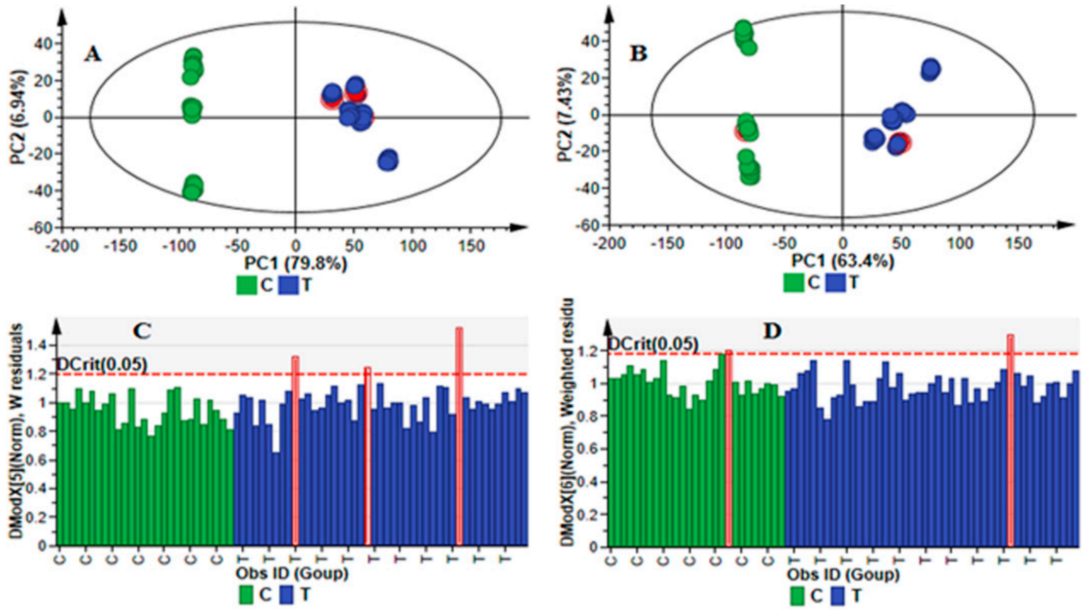

Figure S1. PCA scores and DModX plots (of methods 2 and 3 in Table 2). (**A**) **Scores scatter plot** of the PCA model of the data X (processed with *method 2:* **Table 1**): a 5-component model, explaining 92.6% variation in the *Pareto*-scaled data and the amount of predicted variation by the model, according to cross-validation, is 90.2%. (**B**) **Score scatter plot** of the PCA model of the data X (processed with *method* 3: **Table 1**): a 6-component model, explaining 79.3% variation in the *Pareto*-scaled data X and the amount of predicted variation by the model, according to cross-validation, is 74.4%. (**C**) The **DModX plot** of the PCA model in (A) showing the moderate outliers (in red). (**D**) The **DModX plot** of the PCA model in (B) showing the moderate outliers (in red).

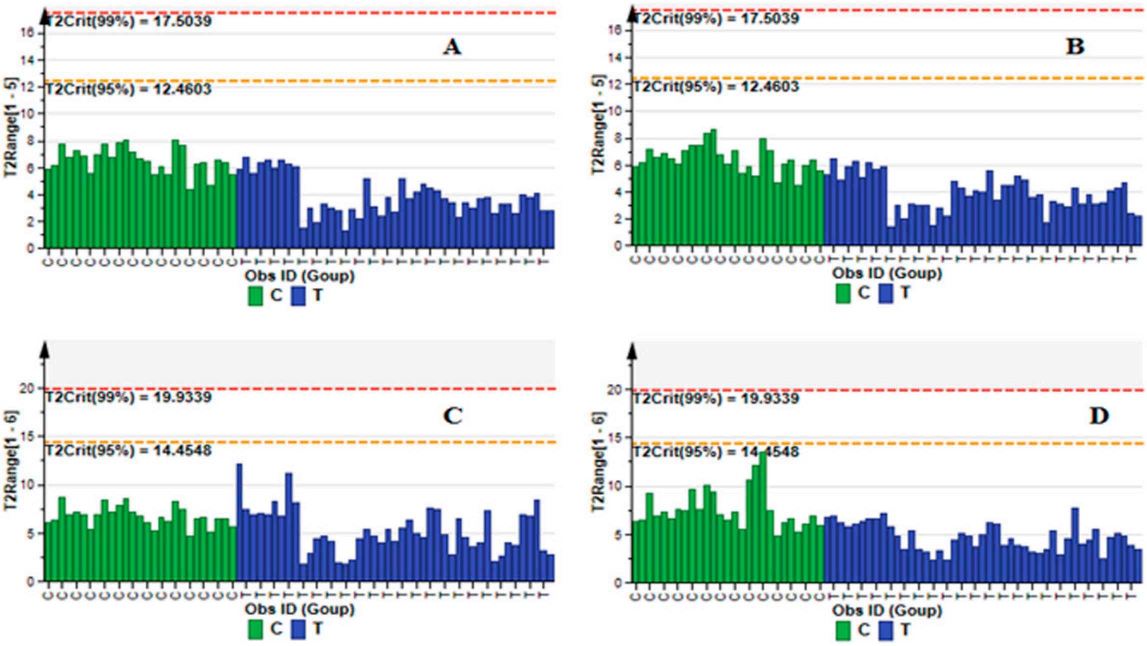

Figure S2. Hotelling's T^2 range plots of the four PCA models (methods 1 to 4 in Table 2). The computed Hotelling's T^2 plots from the first to the last component of the model. The Hotelling's T^2 establishes the "normal" operating area in the model hyperplane (score space), aiding thus in finding the data/samples that deviate strongly from the model (strong outliers). The four plots (**A**, **B**, **C** and **D** for *methods* 1, 2, 3, and 4 respectively) show no strong outliers.

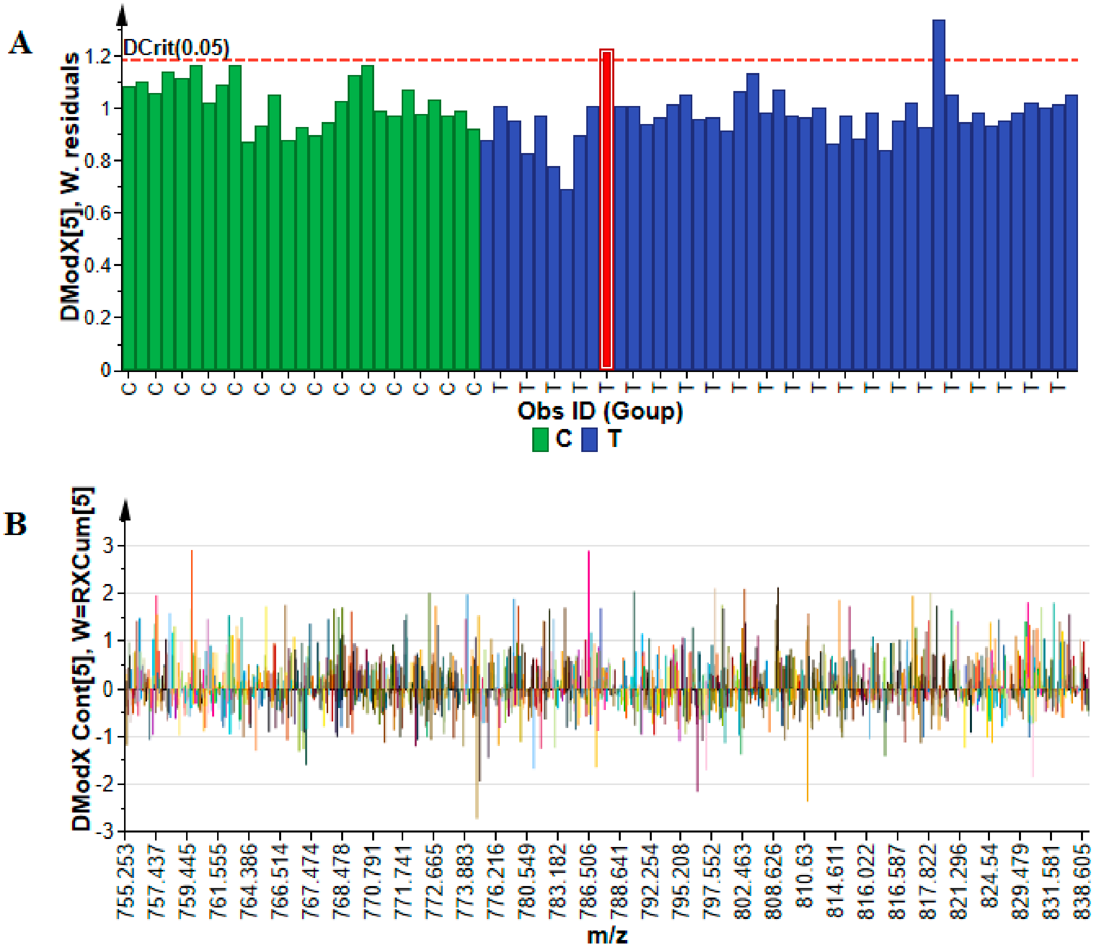

Figure S3. DModX and a typical contribution plots (of the PCA models for the method 1 data set). (**A**) **DModX plot** displaying the moderate outliers. To understand why a sample deviates in DModX, a contribution plot (*e.g.* the plot in **B**) is computed. In this case, the **contribution plot** (**B**) of the selected moderate outlier (red in the DModX plot, **A**) was constructed. The contribution plot displays which variables contribute to the deviation of the selected sample. Rule of thumb is that if there are variables with absolute values above 3 (in the contribution plot, *y*-axis) it means the selected sample needs to be investigated further. In this case, an inspecting the contribution plot (**B**) shows no variable with critical deviation from the rest of the data set.

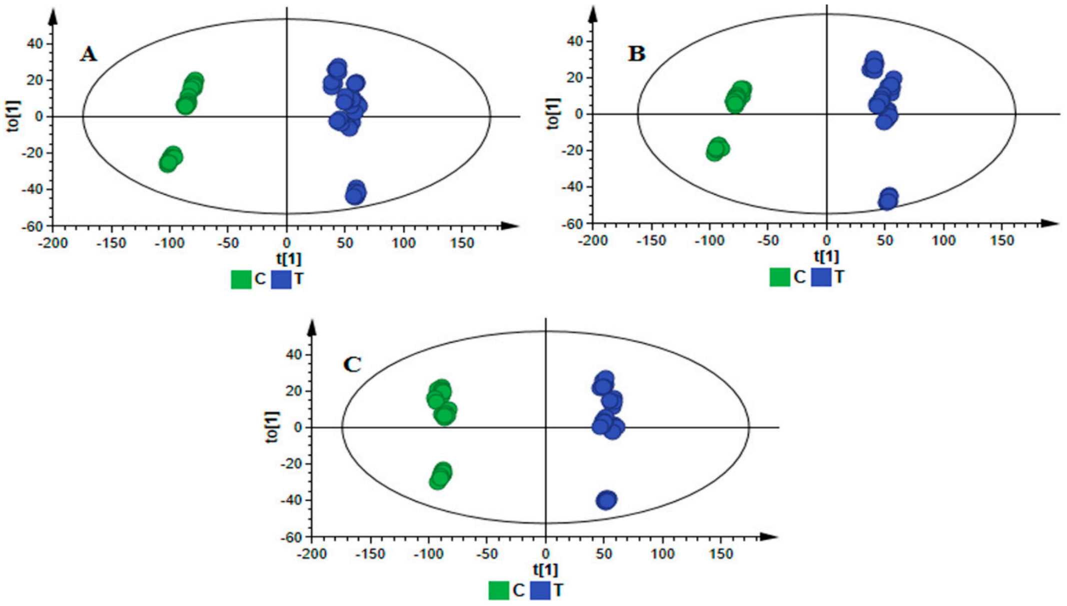

Figure S4. OPLS-DA scores plots. The scores plots for the data sets: *methods 2*, *3* and *4* (**A**, **B** and **C**, respectively) showing clear separation between groups: C (non-treated) and T (treated) samples. The description and quality of the four OPLS-DA models are given **Table 2** (main text).

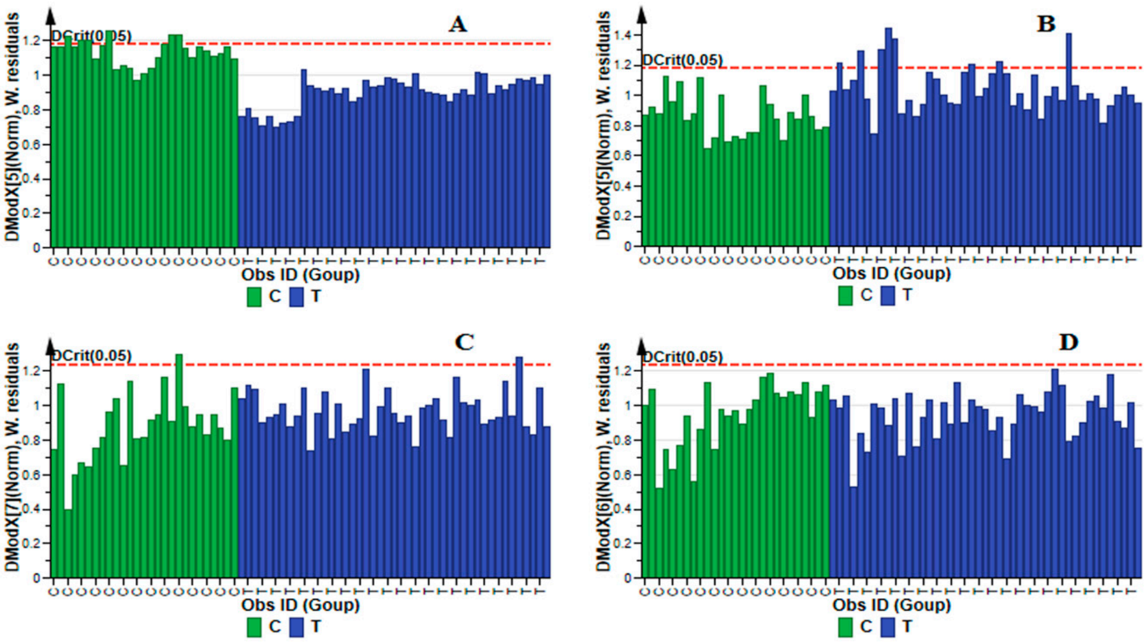

Figure S5. DModX plots for the detection of moderate outliers. The inspection of the DModX plots of PCA models of the same data set but with different data pre-treatments (**A**= UV-scaled, **B** = None-scaling, **C** = Pareto-scaled and log transformation and **D** = UV-scaling and log transformation) show different moderate outliers in different models. This observation illustrates the effect/influence of the data pre-treatment methods on the multivariate models.

Model = Method 1

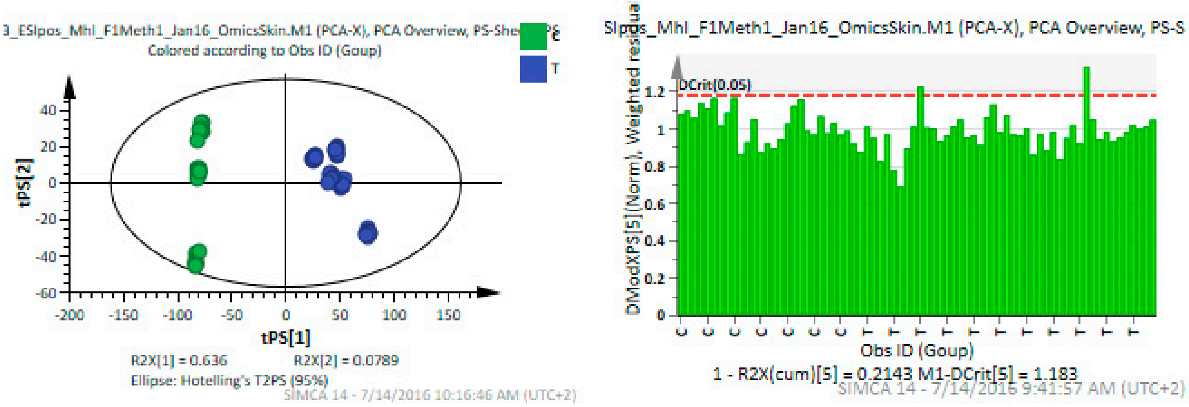

Model = Method 4

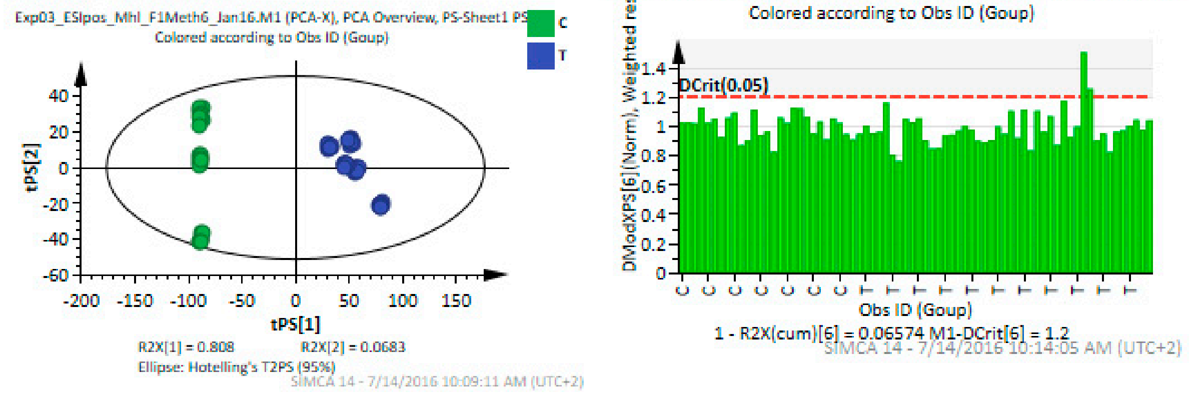

Figure S6. Predicted scores plots and DModXPS. Predicting class membership for all observations in the prediction set assessed the computed SIMCA models. The distance to the model predicted (DModXPS) and the scores plots show how well the prediction set fits the respective SIMCA model. The predicated scores summarize the prediction set observations to the given model. The displayed critical distance (DCrit) corresponds to the 0.05 level and defines a 95% tolerance interval. Here, the selected examples of the predicted scores plots showed clear separation between the two groups (C and T) for all models. Although model quality and description (as shown in Tables 2 and 3) are clearly affected by the pre-processing and pre-treatment methods (as the study points out), it can be concluded that there is separation between the two groups (C and T).

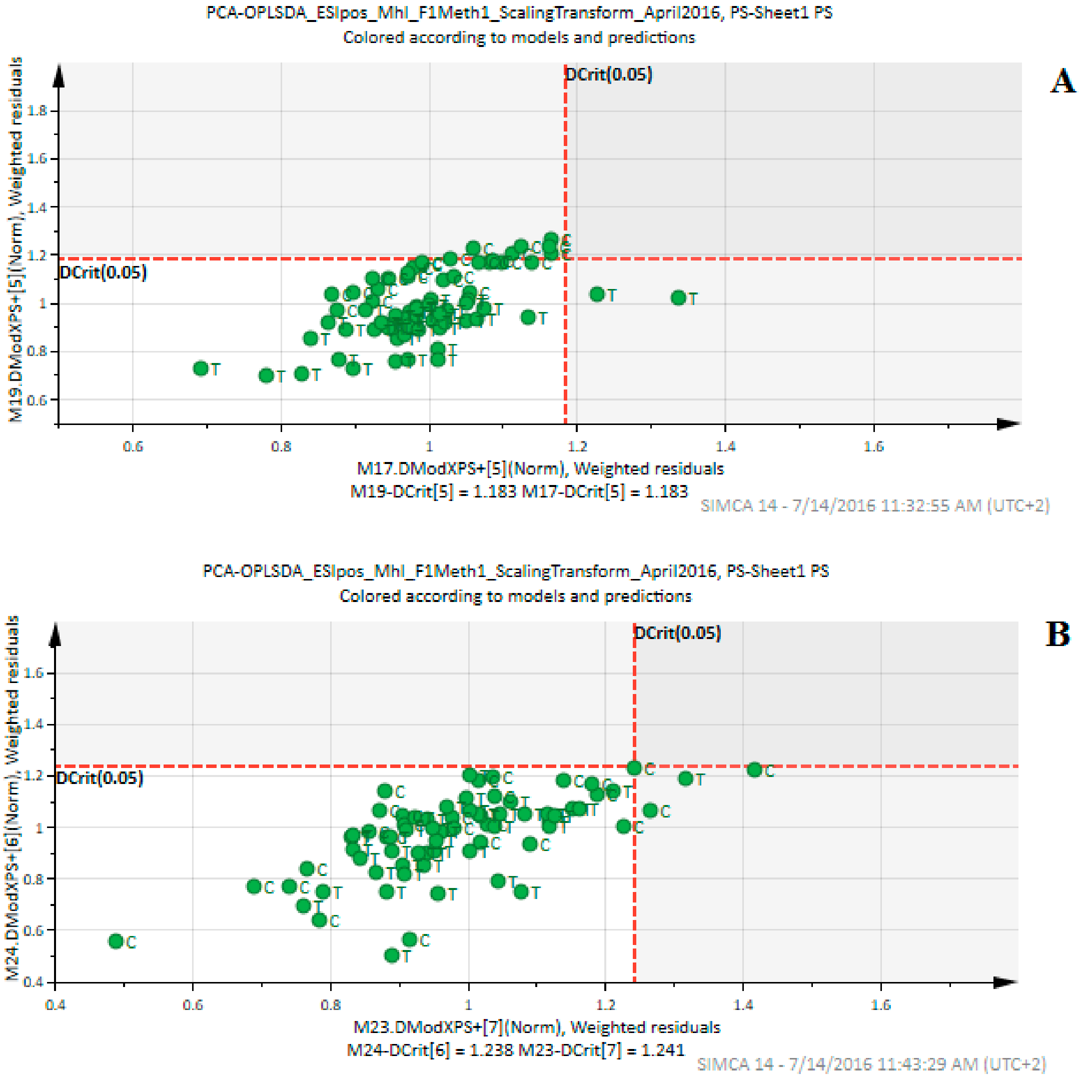

Figure S7. The Coomans' plots - distance to model predicted (DModXPS+) of two models. The Coomans' plot displays DModXPS+ of two models in a scatter plot. It is a classification plot displaying the simultaneous fit of the prediction set observations into two selected models. Left-top corner = samples only fit the model on *y*-axis, right-lower corner = samples only fit the model on *x*-axis, left-lower corner = samples fit both model and right-top corner = samples that are outside both models. (**A**) The Coomans' plot of two models: model of pareto-scaled data (*x*-axis) and model of UV-scaled data (*y*-axis). (**B**) The Coomans' plot of two models: model of pareto- and log transformation data (*x*-axis) and model of UV- and log transformation data (*y*-axis). Although the plots show that majority of the observations fits into both selected models (in two cases), the distribution is different. This observation illustrates also the effect/influence of the data pre-treatment methods on the multivariate models.
